# Human BDNF/TrkB variants impair hippocampal synaptogenesis and associate with neurobehavioural abnormalities

**DOI:** 10.1038/s41598-020-65531-x

**Published:** 2020-06-03

**Authors:** Takuhiro Sonoyama, Lukas K. J. Stadler, Mingyan Zhu, Julia M. Keogh, Elana Henning, Fuki Hisama, Peter Kirwan, Magdalena Jura, Beata K. Blaszczyk, David C. DeWitt, Bas Brouwers, Marko Hyvönen, Inês Barroso, Florian T. Merkle, Suzanne M. Appleyard, Gary A. Wayman, I. Sadaf Farooqi

**Affiliations:** 1 University of Cambridge Metabolic Research Laboratories and NIHR Cambridge Biomedical Research Centre, Wellcome Trust-MRC Institute of Metabolic Science, Addenbrooke’s Hospital, Cambridge, UK; 2 Integrative Physiology and Neuroscience, College of Veterinary Medicine, Washington State University, Pullman, Washington, USA; 3 Department of Medicine (Medical Genetics), University of Washington School of Medicine, Seattle, Washington, USA; 4 Department of Biochemistry, 80 Tennis Court Road, CB2 1QW, University of Cambridge, Cambridge, UK; 5 MRC Epidemiology Unit, Addenbrooke’s Hospital, Cambridge, UK; 6 Wellcome Sanger Institute, Cambridge, UK

**Keywords:** Genetics, Molecular biology, Neuroscience, Endocrinology, Neurology

## Abstract

Brain-derived neurotrophic factor (BDNF) signals through its high affinity receptor Tropomyosin receptor kinase-B (TrkB) to regulate neuronal development, synapse formation and plasticity. In rodents, genetic disruption of *Bdnf* and *TrkB* leads to weight gain and a spectrum of neurobehavioural phenotypes. Here, we functionally characterised a *de novo* missense variant in *BDNF* and seven rare variants in *TrkB* identified in a large cohort of people with severe, childhood-onset obesity. In cells, the E183K BDNF variant resulted in impaired processing and secretion of the mature peptide. Multiple variants in the kinase domain and one variant in the extracellular domain of TrkB led to a loss of function through multiple signalling pathways, impaired neurite outgrowth and dominantly inhibited glutamatergic synaptogenesis in hippocampal neurons. *BDNF/TrkB* variant carriers exhibited learning difficulties, impaired memory, hyperactivity, stereotyped and sometimes, maladaptive behaviours. In conclusion, human loss of function *BDNF/TrkB* variants that impair hippocampal synaptogenesis may contribute to a spectrum of neurobehavioural disorders.

## Introduction

The neurotrophin Brain-Derived Neurotrophic Factor (BDNF) is widely expressed in the mammalian brain and signals via the Tropomyosin receptor kinase B (TrkB) to regulate neuronal differentiation and survival, synapse formation and activity-dependent changes in synapse structure and function. *Bdnf* and *TrkB* null mice are embryonically lethal^1,2^. *Bdnf* haplo-insufficient mice and mice in which *Bdnf* is deleted in the postnatal brain, survive and exhibit hyperactivity, impaired pain sensation, increased food intake and weight gain^3^. In humans, deletions encompassing the *BDNF* gene on chromosome 11p.12.3 and very rare loss of function coding variants in *TrkB* have been reported in individuals with speech and language delay, hyperphagia and severe obesity^4–6^.

BDNF is synthesised as a precursor protein, pre-pro-BDNF, which is converted into pro-BDNF by removal of the signal peptide and packaged into vesicles before being transported distally to dendrites or axons^7^. Only once the protein is destined for secretion, is pro-BDNF converted to mature BDNF through proteolytic cleavage by furin and other proprotein convertases in the trans-Golgi network or secretory vesicles, releasing mature BDNF from the pro-domain^8^. Processing of pro-BDNF and secretion are thought to occur almost simultaneously^9^. The regulated equilibrium between pro-BDNF and mature BDNF appears to be physiologically relevant as a hippocampus-specific deletion of the serine protease tissue plasminogen activator (tPA), which is involved in the cleavage of pro-BDNF to BDNF extracellularly, increases depression and anxiety-like behaviour in adult mice^10^.

Here we functionally characterise a rare coding variant in *BDNF* and several rare variants in *TrkB* recently identified using exome sequencing and targeted sequencing of people with severe obesity^11^. We use these human variants as tools with which to explore the consequences of impaired BDNF-TrkB signalling on dendritic spine structure and function, which forms the neural substrate for learning and memory in hippocampal neurons.

## Results and Discussion

### A rare coding variant in BDNF disrupts processing of pro-BDNF

**Figure 1:**
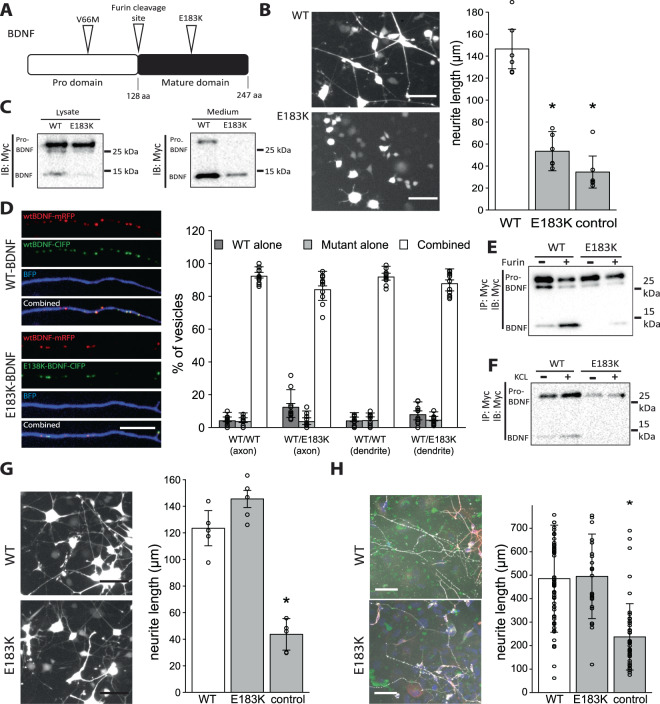
Functional characterisation of a rare coding variant in BDNF (E183K). (**A**). Schematic representation of BDNF protein with the common variant (V66M) and rare variant (E183K) indicated. (**B**). PC12 cells were transfected with WT (top)/E183K (bottom) BDNF; neurite length was measured by fluorescence microscopy. Left panel: representative images from 3 experiments. Scale bar: 50 μm. Average neurite length per nucleus is shown (right panel; data point = mean of replicate); *p < 0.05, student’s t-test. (**C**). WT/mutant BDNF was transfected into PC12 cells and protein quantified by Western blot in cell lysate (left) and growth medium (right) using an antibody against a c-terminally fused myc-tag. (**D**). Cultured primary rat hippocampal neurons were co-transfected with RFP-tagged (red) WT BDNF and ClFP-tagged (green) WT BDNF (top image panel), or RFP-tagged WT and ClFP-tagged mutant (E183K) BDNF (bottom image panel). Co-localisation of the proteins was measured by fluorescent confocal microscopy in axons (shown here) and dendrites, and is presented as proportion of vesicles containing both (combined) or only one of the tagged proteins (center panel; data point = one axon). (Right panel: Density of dendritic BDNF positive vesicles containing either WT/WT BDNF or WT/E186K BDNF) Scale bar: 10 μm. (**E**). WT/mutant BDNF expressed in HEK293 cells was immunoprecipitated, followed by Furin-mediated protein cleavage. The cleavage products were analysed by Western blot. (**F**). WT/mutant BDNF were transfected into PC12 cells and depolarisation-dependent BDNF secretion triggered by addition of KCl. Amounts of secreted BDNF were measured by Western blotting. (**G**). TrkB-expressing PC12 cells were transfected with GFP and stimulated with synthetic WT/mutant BDNF. Neurite outgrowth was assessed by fluorescent microscopy (left panel; scale bar: 50 μm); average neurite length per nucleus shown in right panel (data point = mean of replicate). *p < 0.05, student’s t-test. H. Human iPSCs were differentiated into hypothalamic POMC-expressing neurons and stimulated with synthetic WT/mutant BDNF. Neurite outgrowth was assessed by microscopy (left panel; scale bar: 100 μm). Shown are average neurite length per nucleus (right panel; data point = one cell); *p < 0.05, student’s t-test.

While several common variants in BDNF exist (including the widely studied variant p.V66M; variant allele frequency: 19%), to date, no rare coding variants in this gene have been reported. Here, we identified a single heterozygous missense variant in BDNF (p.E183K) in a 15 year old girl with severe obesity and moderately severe learning difficulties (Fig. 1A; Table 1). This variant was not reported in publically available databases (http://gnomad.broadinstitute.org/); it was inherited from her father (BMI 36 kg/m^2^) who also had learning difficulties. We performed a number of experiments to test whether this variant had functional consequences in cells.

**Table 1:** Neurobehavioural phenotypes seen in carriers of rare variants in *BDNF* and *NTRK2*.

Variant	Age (years)	Gender^A^	BMI (sds)	Hypotonia	Learning difficulties (S)	Dyspraxia	Hyperactivity/inattention	Aggression	Anxiety	Impaired Memory	Altered Nociception	Other
BDNF												
E183K	15.5	F	33 (2.9)	O^B^	O			O				
TrkB												
P204H^C^	3.6	F	31 (5.5)		O S^D^	O	O	O		O	O	Impaired thermoregulation
P660L	6.6	M	24 (3.5)	O	O	O						autistic traits
R691H	9.3	F	27 (2.9)			O		O			O	Impaired thermoregulation
R696K	15.9	F	42 (3.8)		O							
S714F	33	F	60		O							
R715Q	2.6	F	37 (6.7)	O	O S		O	O	O	O	O	reduced foetal movements; fearless
R715Q	10.1	M	27 (3.0)	O	O S	O	O	O				autistic traits
R715W	18.5	F	50		O							
Y722C	7.5	M	32 (4.3)	O	O S	O	O	O		O	O	absence seizures; respiratory difficulties; autistic traits
T821A	3.9	M	28 (5.3)	O	O S							
P831L	15.1	F	34 (3.1)	O	O S	O	O	O		O	O	respiratory difficulties

^A^M: male; F: female

^B^‘O’ reported presence of a phenotype where known

^C^The patient carrying P204H in *NTRK2* (encoding TrkB) was found to harbour a pathogenic mutation in *GNAS*.

^D^‘S’ speech and language delay

While mature BDNF can be secreted constitutively, it is preferentially released via a tightly controlled regulated pathway driven by activity-dependent depolarisation and Ca^2+^ entry^12^. We first tested whether the E183K BDNF variant had any functional impact on BDNF globally, by transfecting wild-type (WT) and mutant BDNF into PC12 cells, which stably express human TrkB (PC12^TrkB^), followed by measurement of neurite outgrowth 48 hours later. We found that mutant BDNF was significantly impaired in its ability to stimulate neurite outgrowth compared to WT (Fig. 1B, n = 4, p < 0.05), demonstrating that the E183K substitution causes a loss-of-function. Expression levels of WT and E183K pro-BDNF in cell lysates were comparable (Fig. 1C). To test if packaging of the protein into vesicles or distal trafficking might be affected, primary rat hippocampal neurons were co-transfected with differentially labeled WT and E183K mutant BDNF. After 48 hours, protein localisation was measured using confocal-fluorescent microscopy and found to be strongly overlapping in dendrites and axons (Fig. 1D), while vesicular density was reduced when WT and mutant constructs were co-expressed (Figure S1A, n = 3, p < 0.05). These results suggest that although packaging of the mutant pro-BDNF into vesicles is reduced, trafficking of E183K BDNF to the distal parts of the neuron can occur. We next tested proteolytic cleavage of mutant pro-BDNF by capturing epitope-tagged BDNF on antibody-coated beads, followed by incubation with purified, recombinant furin. We found that, while WT pro-BDNF was efficiently processed to the mature form, E183K disrupted pro-BDNF processing by the protease (Fig. 1E). Thus defective processing of the pro-form is the most likely explanation for the significantly reduced levels of mature mutant vs WT BDNF detected in cell lysates, and likely a contributing factor to the same observation in cell culture medium, where defective secretion may also play a role (Fig. 1C). It is not clear how the mutation at position 183 could disrupt furin-mediated cleavage at position 128. However, it is conceivable that in the tertiary structure of the pro-peptide those two positions are proximal to each other (no structural data are available). Next, we tested regulated secretion of WT vs mutant BDNF in PC12 cells following depolarisation by potassium chloride (90 mM KCl). KCl treatment led to the detection of increased amounts of immature and mature BDNF in the medium of cells transfected with WT - but not with mutant - BDNF, thus confirming that the E183K variant impairs regulated BDNF secretion (Fig. 1F).

Once secreted, mature BDNF binds with high affinity to TrkB and with much lower affinity to the p75 neurotrophin receptor^13^. Upon ligand binding, TrkB auto-phosphorylates several tyrosine residues within its tyrosine kinase domain to initiate signalling via the Mitogen-Activated Protein Kinase (MAPK), Phosphoinositide 3-kinase (PI3K) and Phospholipase C (PLC)-γ pathways. Signalling via PLCγ and PI3K regulates synaptic plasticity and neuronal survival respectively, while MAPK promotes neuronal maturation and differentiation^14,15^. We explored the effect of WT/E183K BDNF on downstream signalling and neurite outgrowth by applying chemically synthesized mature WT/E183K BDNF to PC12^TrkB^ (Figure S1B). We observed no significant difference in the activation of any of the three major signalling pathways downstream of TrkB (Figure S1C) or in the mutant’s ability to induce neurite outgrowth in PC12^TrkB^ (Fig. 1G). Furthermore, in human stem-cell derived hypothalamic POMC neurons, cells which are directly involved in energy homeostasis, WT and mutant BDNF-mediated neurite outgrowth and cellular migration were comparable (Fig. 1H, S1D). In conclusion, the E183K substitution has no functional consequences on the mature peptide, but impairs processing, trafficking and secretion of the pro-hormone (Fig. 1B,C,D).

### Rare variants in NTRK2 which encodes TrkB disrupt signalling and neurite outgrowth

**Figure 2:**
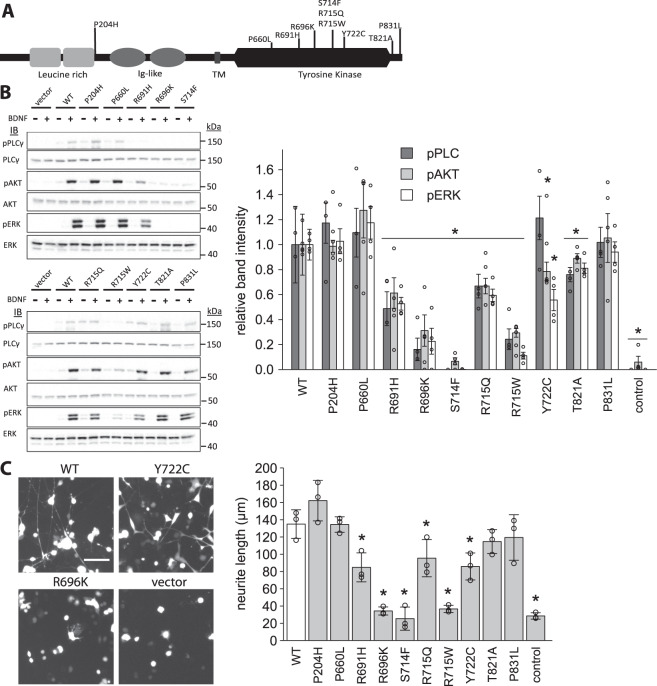
Functional characterisation of TrkB mutants. (**A**). Schematic representation of TrkB protein with rare variants indicated; Immunoglobulin (Ig)-like domain; TM (transmembrane region). (**B**). PC12 cells were transiently transfected with WT/ mutant TrkB. Phosphorylation of downstream signalling molecules was measured by Western Blot before (−) and after (+) stimulation with recombinant BDNF; band intensities quantified by densitometry (right panel; data point = signal from one replicate). Mutants are expressed relative to WT. *p < 0.05, student’s t-test. (**C**). PC12 cells were transiently transfected with WT/mutant TrkB as well as GFP and stimulated with recombinant BDNF for 48 h. Neurite length was measured by fluorescent microscopy (representative images from 2 mutants shown in left panel; remainder shown in Figure S2) and quantified as length per nucleus (right panel; data point = mean of one replicate). Scale bar: 50 μm. *p < 0.05, student’s t-test.

We next functionally characterised seven missense variants in TrkB (encoded by the *NTRK2* gene) as well as three previously reported variants (Y722C, P660L, T821A)^5,16^. All variants were found in heterozygous form; one variant (R715Q) was found in 2 unrelated individuals (Table 1). TrkB is composed of 821 amino acid residues that encode a signal peptide, two cysteine-rich clusters, a leucine-rich motif, two immunoglobulin-like C2-type motifs, a transmembrane region and the canonical tyrosine kinase domain, which is required for signalling (Fig. 2A). Of the ten variants investigated, eight lie in the tyrosine kinase domain, one at the very c-terminal end of the protein, and one in the extracellular domain of the protein (Fig. 2A). None of the TrkB variants reduced protein expression (Figure S2A). We transiently transfected PC12 cells (which do not endogenously express TrkB) with WT/mutant TrkB. After stimulation with recombinant BDNF, cells were lysed and phosphorylation of the three downstream effectors (PLCγ, AKT, ERK) measured using Western blotting (Fig. 2B). All five of the newly identified kinase domain variants resulted in significantly impaired signalling through all three pathways (n = 4, p < 0.05). The variants in the extracellular domain (P204H) and at the C-terminus (P831L) did not affect function in these assays. The same five mutants also led to a significant reduction in neurite outgrowth, with the level of impairment reflecting the extent of reduced signalling (Fig. 2C, Figure S2B, n = 3, p < 0.05). Additionally, all variants in the kinase domain – with the exception of the previously reported P660L – led to significantly diminished cell survival under serum starved conditions (Figure S2C; n = 3, p < 0.05). Taken together, the five newly identified TrkB kinase domain variants result in a significant loss-of-function in cells. Of interest, four of those five variants (R691H, R696K, R715Q, R715W) affect Arginine residues, which are situated at the active site of the kinase domain and likely play a role in catalysis^17^. The fifth variant (S714F), has the most disruptive effect of all mutations tested here, and falls immediately adjacent to the highly conserved DFG motif, which is indispensable for kinase activity.

### Loss of function TrkB variants affect glutamatergic synaptogenesis

**Figure 3:**
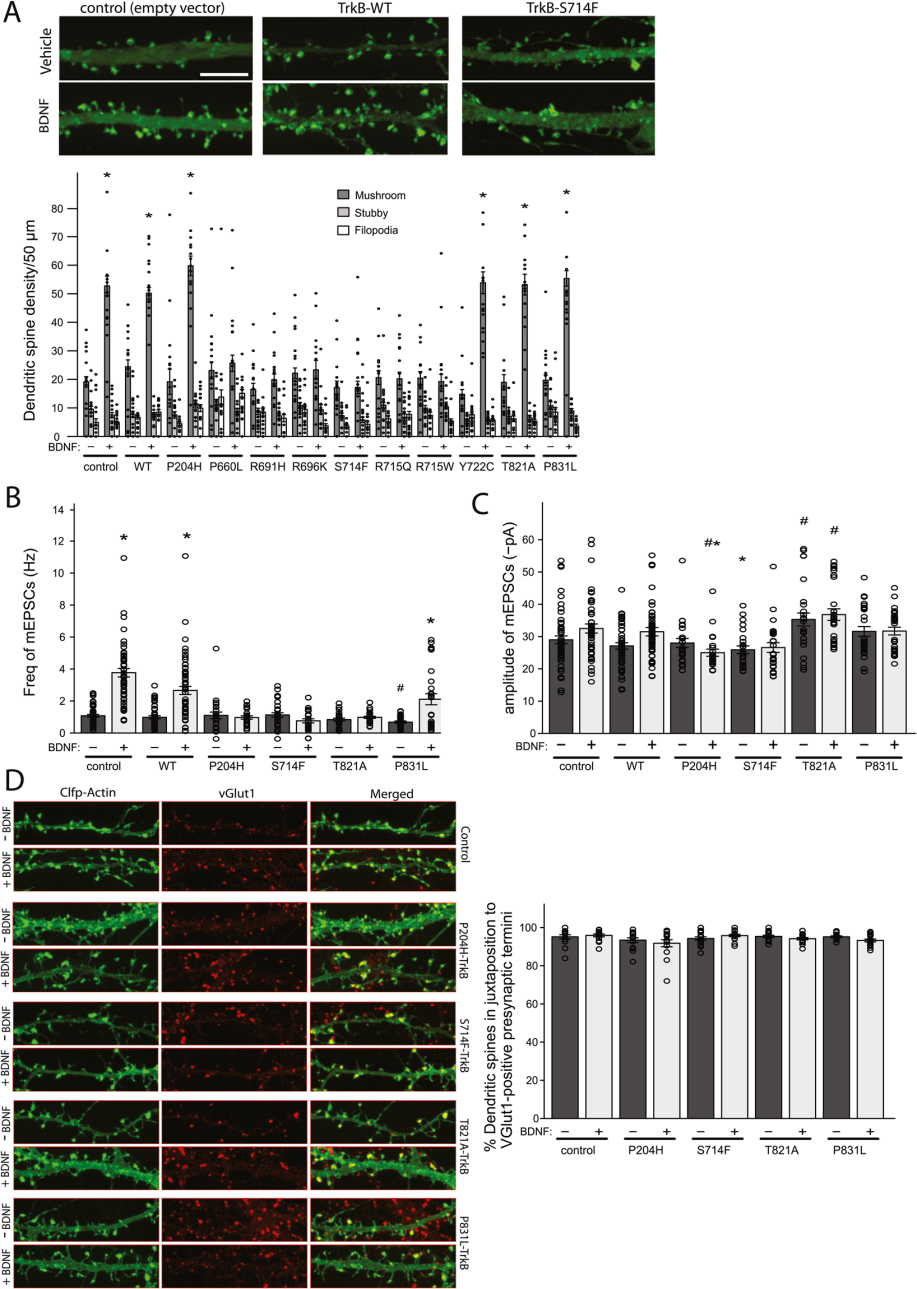
Functional characterisation of TrkB mutants in neurons. (**A**). Primary dissociated hippocampal neurons were transfected with WT/mutant TrkB alongside Clover fluorescent protein (ClFP)-tagged actin and stimulated with either vehicle or recombinant BDNF. Dendritic spine density was assessed by confocal microscopy (upper panel: images from one mutant (S714F) shown. Scale bar: 10 μm); lower panel includes data from all mutants (number of spines per 50 μm of dendrite length; data point = one dendrite). *p < 0.01, student’s t-test, ± SEM. (**B**). Patch-clamp experiments were performed on rat hippocampal neurons transiently transfected with WT/mutant TrkB, before (−) and after (+) recombinant BDNF. Shown is the frequency of mEPSCs for all cells recorded. *indicates significant (p < 0.01) increase compared to unstimulated state; by one-way ANOVA-Tukey, F = 28.93. ^#^indicates significant (p < 0.05) decrease compared to unstimulated control (baseline); by one-way ANOVA-Tukey, F = 15.4 ± SEM. (**C**). Shown is amplitude of mEPSCs for all cells recorded. *indicates significant (p < 0.05) reduction compared to BDNF-stimulated control; by one-way ANOVA-Tukey, F = 8.024. ^#^indicates significant (p < 0.05) difference to unstimulated control; by one-way ANOVA-Tukey, F = 10.03. (**D**). Analysis of pre and post-synaptic correlation was done by transfection of ClFP-Actin (to highlight dendritic spines) and WT/mutant TrkB followed by immunostaining of presynaptic terminals with anti-vGlut1 before (−) and after (+) recombinant BDNF addition to DIV11. Representative images are shown in left panel and percentage of dendritic spines (stubby and mushroom) positive for vGlut1 positive presynaptic terminals is shown in right panel ± SEM.

BDNF and TrkB are highly expressed in areas of the brain involved in learning and memory including the hippocampus. BDNF expression within the developing hippocampus also coincides with a period of rapid glutamatergic synaptogenesis^18–20^. Dendritic spines are the main sites of excitatory synaptic input for neurons; as such factors that affect their density and morphology can alter synaptic transmission and neuronal plasticity^21^. Several forms of learning can increase the number of dendritic spines^22–24^, for example, spatial learning is associated with an increase in hippocampal spine densities^25^, whereas aging leads to a decline in hippocampal spine densities. Experiments in hippocampal neurons have shown that activity-driven local synthesis and release of mature BDNF controls dendritic spine density and morphology, long-term potentiation and the plasticity of dendritic spines^26^. To test the effects of human TrkB variants on these processes, we isolated primary neurons from rat hippocampus and tested BDNF’s ability to promote the formation of dendritic spines following transfection with either WT or mutant TrkB. A dendritic spine can change its shape within a short time from a thin spine with a small head and a narrow neck (filopodia; the least developed) to a dendritic spine with a large head and a narrow neck (mushroom spines; the most developed) as well as ‘stubby’ spines, which display a transitional morphology^27^. To investigate the ability of TrkB mutants to support BDNF stimulated spinogenesis, we transfected primary rat hippocampal neurons, which endogenously express TrkB, with various recombinant wt or mutant human TrkB receptor mutants (Fig. 3). BDNF treatment resulted in a selective increase in mushroom spines (Fig. 3A, n = 4, p < 0.01), but had no significant effect on the density of filopodia or stubby spines, suggesting that TrkB signaling is involved in the maturation process to mushroom spines. Overexpression of WT human TrkB had no significant effect on the density of mushroom spines, however expression of most of the kinase domain mutants (P660L, R691H, R696K, S714F, R715Q and R715W) completely abolished the spine-stimulatory effect of BDNF. Interestingly, this observation is strongly indicative of a dominant-negative effect exerted by the mutant receptor over the endogenous WT receptor in these neurons, a hypothesis that will require additional experiments to test. These findings align with work from Rauskolb and colleagues who found that a decrease in mature BDNF in the rodent hippocampus reduces mature mushroom type spines and increases less stable thin spines^28^. An inhibition in the number of mature mushroom shaped dendritic spines strongly suggests that these mutants suppress the ability of BDNF to stimulate synaptogenesis. To test whether the changes were associated with functional changes, we investigated the effect of TrkB mutants on BDNF-stimulated increases in miniature excitatory synaptic currents (mEPSC). We found that BDNF increased mEPSC frequency in either control (vector only) transfected neurons or neurons transfected with WT TrkB, (Fig. 3B and Figure S3, n = 3, p < 0.01), but did not affect amplitude (Fig. 3C). Taken together with the increase in spines, this suggests BDNF-TrkB signaling increases functional synapses, as has been reported previously^14^.

We then went on to test the effect of a subset of TrkB variants on mEPSC frequency and found that the loss-of-function TrkB variant S714F abolished the BDNF-stimulated increase in mEPSC frequency (Fig. 3B, S3). Interestingly, P204H and T821A also blocked, while P831L greatly attenuated, BDNF’s effect to increase mEPSC frequency (Fig. 3B). This is in contrast to their lack of effect on spine number suggesting these mutants affect the maturation of spines into functional synapses, rather than spine formation. Similar to the effect on dendritic spines described above, the mutants had a dominant negative effect, as the ability of endogenous TrkB to increase mEPSC frequency was abolished by overexpression of the mutants. BDNF did not significantly change the amplitude of the mEPSCs following transfection of any of the TrkB mutations compared to their own controls (when standardised relative to the unstimulated control group, Fig. 3C). However, the average amplitude of mEPSCs was very slightly, but significantly, higher in neurons expressing the T821A mutation compared to control neurons, suggesting that this mutation may alter the basal expression or function of AMPA receptors (AMPA: α-amino-3-hydroxy-5-methyl-4-isoxazolepropionic acid) (Fig. 3C, n = 3, p < 0.05).

**Figure 4:**
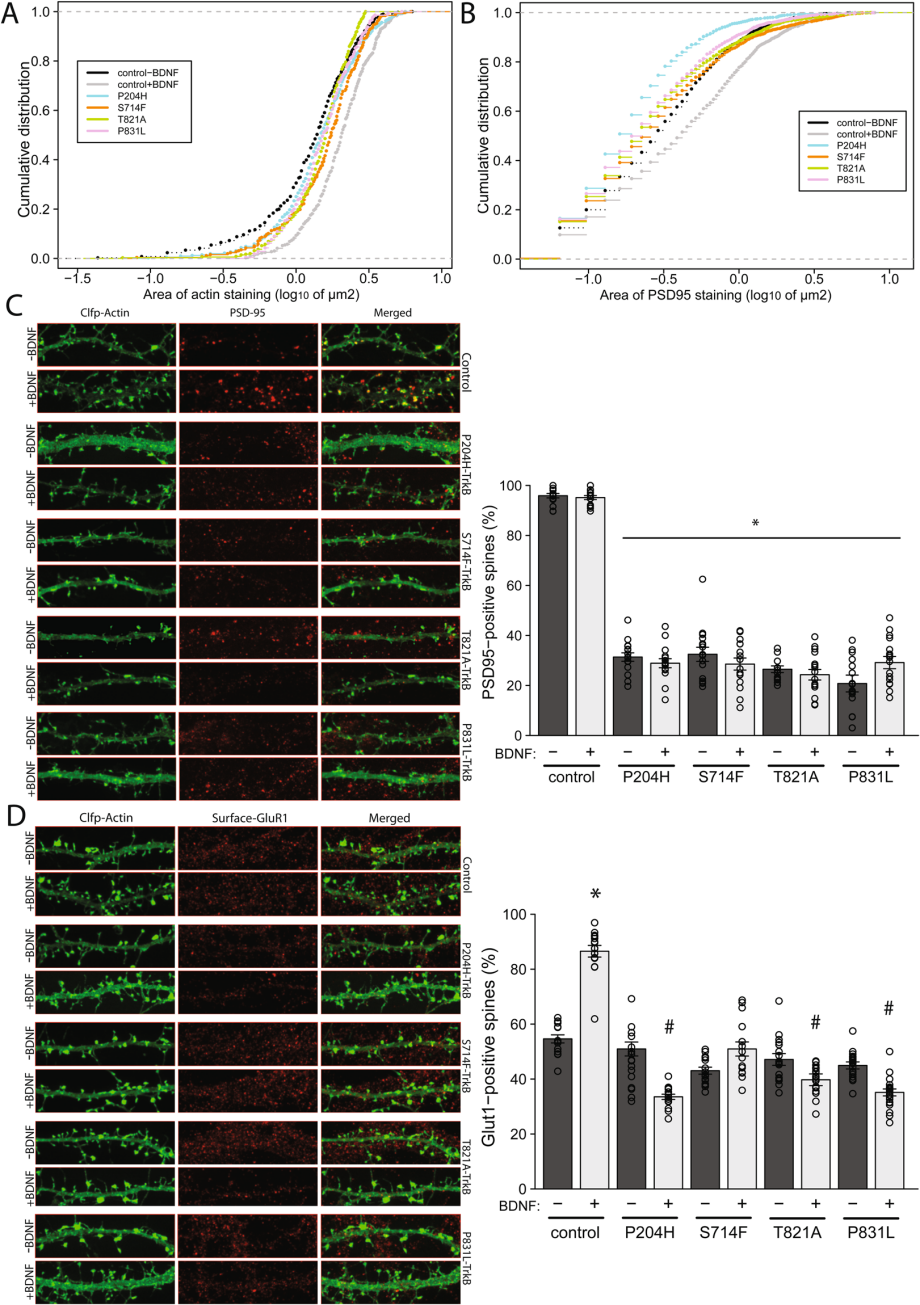
Effects of TrkB mutants of on dendritic spine maturation. (**A**). To determine the effects of TrkB mutants on dendritic spines size, rat hippocampal neurons - transiently transfected with Clover fluorescent protein-actin and WT/mutant TrkB - were analysed for spine area before (−) and after (+) recombinant BDNF on DIV11. Representative images are shown in Figs. 3C and 4C (images used for quantification). Cumulative frequency plots for control + /− BDNF and mutants + BDNF are shown. In control, stimulation with BDNF (grey vs black), significantly increases the size of spines. p < 2.2*10^−16^, Kolmogorov–Smirnov (K-S) test. Expression of mutant forms of Trkb stunts BDNF’s ability to promote spine growth (colours vs grey). p < 0.001, K-S test. (**B**). To determine the effect of TrkB mutants on post synaptic density sizes, rat hippocampal neurons were transiently transfected with ClFP-Actin and WT/mutant TrkBs (DIV5) and stimulated with vehicle or BDNF (DIV7-11), followed by staining for PSD-95. Representative images are shown (Fig. 4C). Cumulative frequency plots for control + /− BDNF and mutants + BDNF are shown. In control, stimulation with BDNF (grey vs black), significantly increases the area if PSD-95 staining. p < 3*10^−15^, K-S test. Expression of mutant Trkb diminishes BDNF’s stimulatory effect on postsynaptic density size (colours vs grey). p < 2.2*10^−16^, K-S test. (**C**). PSD-95 staining of dendritic spines (mushroom and stubby) is shown in the left-hand panel and proportion of spines positive for PSD-95 staining following expression of WT/mutants is shown in the right-hand panel. PSD-95 staining is reduced for all mutants in the stimulated and unstimulated state. *p < 0.01, student’s t-test ± SEM. (**D**). To determine the effects of TrkB mutants on the surface expression of GluR1-containing AMPA receptors in dendritic spines, rat hippocampal neurons were transiently transfected with ClFP-actin and WT/mutant TrkBs, followed by live staining for GluR1 in the presence or absence of recombinant BDNF (DIV7-11). Representative images are shown (left-hand panel) and the percentage of dendritic spines (mushroom and stubby) positive for surface GluR1 staining is shown in the right-hand panel. In control, stimulation with BDNF significantly (*p < 0.01, student’s t-test ± SEM) increases GluR1 staining in spines, but shows the reverse effect (i.e. decrease) in 3 of the 4 mutants tested (^#^p < 0.05, student’s t-test ± SEM).

To further test if the P204H, S714F, T821A, and P831L mutations change spine maturation and synapse formation, we next analysed synaptic structure and Glutamate Receptor 1 (GluR1) surface expression. We first addressed whether these mutants altered the formation of “morphological” synapses, i.e. if the dendritic spines failed to make connections with presynaptic axonal terminals. We transfected hippocampal neurons with the various mutant TrkBs and ClFP-Actin to identify mature dendritic spines (mushroom and stubby). We then stained for vesicular Glutamate transporter 1 (vGlut1) to identify glutamatergic presynaptic termini and determined pre- and post-synaptic correlation. Expression of the mutants had no apparent effect on the proportion of dendritic spines that had an associated presynaptic terminal (Fig. 3D), with pre- and post-synaptic correlation ≈ 95% under both control and BDNF-stimulated conditions. This suggests that the mutations in the TrkB receptor do not alter the recruitment of the presynaptic terminals to dendritic spines. As the size of the dendritic spine has been associated with synaptic efficacy, we next determined the effect of these mutants on the size distribution of dendritic spines (mushroom and stubby). As before, mutant TrkBs were expressed with ClFP-actin, then the area of the dendritic spine was measured. We found that BDNF stimulation significantly increased the area of the dendritic spine compared to untreated conditions (Fig. 4A, n = 3, p < 2.2*10^−16^). Interestingly, expression of the four TrkB mutants tested significantly reduced the ability of BDNF to increase overall spine size (Fig. 4A, n = 3, p < 0.001), as well as average spine size (unstimulated: 1.533 ± 0.047 μm^2^, BDNF: 2.238 ± 0.053 μm^2^), P204H (unstimulated: 1.152 ± 0.036 μm^2^, BDNF: 1.740 ± 0.101 μm^2^), S714F (unstimulated: 1.634 ± 0.59 μm^2^, BDNF: 1.851 ± 0.119 μm^2^), T821A (unstimulated: 1.714 ± 0.052 μm^2^, BDNF: 1.677 ± 0.677 μm^2^), P831L (unstimulated: 1.712 ± 0.052 μm^2^, BDNF: 1.699 ± 0.119 μm^2^); n = 3 independent cultures, 14-16 neurons analysed, 675–1180 spines/data point). To further characterise the dendritic spines, we also examined the expression of the postsynaptic and AMPA-GluR1 anchoring protein PSD-95 (postsynaptic density protein 95). Under both unstimulated and BDNF stimulated conditions ≈95% of dendritic spines (mushroom and stubby) of control neurons were positive for PSD-95 (Fig. 4C). In contrast, only ≈30% of dendritic spines were positive for PSD-95 in neurons expressing the TrkB mutants, P294H S714F, T821A and P831L (Fig. 4C). Moreover, the size of the spine area covered by PSD-95 staining was significantly increased by BDNF treatment in control neurons (Fig. 4B, n = 3, p = 3*10^–15^), an effect that was diminished when expressing any of the four TrkB mutants (Fig. 4B, n = 3, p < 2.2*10^−16^).

Synaptic efficacy can also be influenced by the amount of surface expressed AMPA receptors^29^. PSD-95 is a key scaffolding molecule for AMPA receptors, especially those containing GluR1 subunits, and BDNF regulates the trafficking of the AMPA glutamate receptor in hippocampal neurons^30,31^. We therefore examined the expression of surface expressed GluR1. Under control conditions ≈55% of dendritic spines were positive for GluR1. BDNF stimulation increased this to ≈87% (Fig. 4D, n = 4, p < 0.01). Expression of mutant TrkB receptors blocked the BDNF-induced increases in the number of dendritic spines positive for GluR1. In fact, the proportion of dendritic spines positive for GluR1 actually decreased upon BDNF-treatment in neurons expressing P204H, T821A and P831L (Fig. 4D, n = 3, p < 0.05). This apparent decrease is most likely due to BDNF increasing spinogenesis in the presence of those TrkB mutants, but these newly formed spines do not express surface GluR1s (Figs. 3 and 4). Taken together our data support the conclusion that expression of P204H, T821A and P831L TrkB does not block the initial event of spinogenesis, e.g. spine formation, but rather blocks spine maturation, GluR1 membrane insertion and functionality.

### BDNF and TrkB variant carriers exhibit hyperactivity, maladaptive behaviours and impaired short-term memory

We sought to obtain clinical data on variant carriers where possible. In addition to severe obesity, variant carriers exhibited a spectrum of learning difficulties, impaired short-term memory, hyperactivity, repetitive behaviours often considered to be autistic-like, fearlessness and in some cases aggression (Table 1). Some of these neurobehavioural phenotypes as well as increased locomotor activity have been observed in animal models of *Bdnf/Trkb* disruption^2,3^. Whilst playing an important developmental role, deletion of this pathway in mature mice also leads to impaired function^3^, in keeping with BDNF’s role in neuronal maintenance and cell survival shown here.

In summary, we demonstrate that loss of function human variants in BDNF/TrkB are associated with a spectrum of neurobehavioural phenotypes and affect BDNF-stimulated synaptogenesis, particularly maturation of a functional synapse, in hippocampal neurons. By studying rare variants that caused a severe loss of function, we were able to demonstrate effects on signalling, neurite outgrowth and glutamatergic synapse formation and function. The effects we observe with rare, highly penetrant variants in BDNF and TrkB support the view that more common genetic variants that have a modest effect on BDNF/TrkB secretion or signalling may contribute to a spectrum of neurobehavioural phenotypes by affecting hippocampal synaptogenesis. A common variant in BDNF (V66M), found in heterozygous form in approximately 20% of the population, has been shown to reduce activity-dependent secretion of mature BDNF^32^. In human experimental studies, V66M BDNF heterozygous carriers have episodic and long-term memory deficits^33^, reduced hippocampal blood flow during memory tasks and reduced hippocampal volumes measured by MRI. In larger association studies, V66M has been associated with a number of traits and neuropsychiatric disorders including anxiety and depression^34,35^. Therapeutic strategies that lead to an increase in BDNF levels and/or signalling may have beneficial effects on neuronal plasticity, learning, memory and other behavioural phenotypes.

## Methods

### Study design and approval

All genetic and clinical studies were approved by the Research Ethics Service (RES) Committee East of England- Cambridge South (The Old Chapel Royal Standard Place Nottingham, NG1 6FS) and conducted in accordance with the Declaration of Helsinki. Written informed consent was obtained from all subjects or, if subjects were under 18 years of age, from a parent and/or legal guardian. Variant carriers were identified as part of genetic studies of individuals recruited to the Genetics of Obesity Study (GOOS), a cohort of 7,000 individuals with severe early-onset obesity; age of obesity onset less than 10 years. Severe obesity is defined as a Body Mass Index (weight in kilograms divided by the square of the height in meters) standard deviation score more than or equal to 3 (standard deviation scores calculated according to the United Kingdom reference population). Exome sequencing and targeted resequencing was performed in 2,548 European ancestry individuals of the GOOS cohort and in 1,117 ancestry-matched controls as reported previously^11^.

### Statistical analysis of data

All experiments were repeated completely independently of each other at least 3 times (n = x indicated in the main text). For each experiment number of cells counted/measured is stated in the relevant Methods subsection. Acceptance/Rejection of the H0 for all normally distributed data was based on student’s t-test or Anova-Tukey, as indicated in the figure legends. For continuous probability distributions the Kolmogorov–Smirnov test was used.

### Plasmids and mutagenesis

Human cDNA clones of TrkB (RC221794) and BDNF (RC212882) with C-terminal Myc- and Flag-tags were obtained from Origene (Rockville, MD, USA) within the pCMV6-entry backbone. For studies in hippocampal neurons BDNF and TrkB mutants were subcloned into pCAGGS vectors using the Gateway system. In order to introduce patient mutations, site directed mutagenesis was performed using the QuickChange II Kit (Agilent Technologies, CA, USA) according to the manufacturer’s instructions.

### Cells

PC12 cells were obtained from Harvey McMahon (MRC, Cambridge, UK) and TrkB-expressing PC12 cells (PC12TrkB cells) were obtained from Linagyou Rui (University of Michigan). Both cell lines were maintained in RPMI1640 supplemented with 7.5% fetal bovine serum and 7.5% horse serum. HEK293 cells (Sigma, cat no: 85120602) were cultured in DMEM supplemented with 10% fetal bovine serum.

### TrkB signalling assay

TrkB-expressing PC12 cells were serum-starved for 24 hours using serum-free RPMI1640 followed by stimulation with BDNF (RnD Systems, UK) at 50 ng/ml for 10 minutes. Cells were washed once with ice cold PBS and then lysed with lysis buffer (150 mM NaCl, 50 mM Tris pH7.4, 1% Triton) supplemented with the Complete Mini protease Inhibitor Cocktail (Roche, Basel, Switzerland). The cell lysate was then centrifuged for 10 minutes at 13,000 rpm and the supernatant stored at −80 ^o^C or prepared for Immunoblotting.

### Polyacrylamide gel electrophoresis and Immunoblotting

Before gel electrophoresis the cell lysate was supplemented with Bolt reducing agent and Bolt gel loading buffer (both Thermo Fisher Scientific, MA, USA) and incubated at 90 °C for 5 minutes. The samples were then separated by SDS-PAGE (Bolt Gels, Thermo Fisher Scientific, MA, USA), followed by transfer onto nitrocellulose membrane, which were blocked in 3% skimmed milk in PBS + 0.1% Tween (PBS-T) for 1 hour at room temperature. Immunoblotting was performed at 4 °C overnight using the following primary antibodies diluted in blocking buffer: phospho/total p44/42 MAPK, phospho/total Akt, phospho/total PLCgamma1 (all at 1:1000 and all from Cell Signaling Technology, USA), or anti-Myc-tag (1:1000; Merck Millipore, MA, USA). The following day the membrane was washed three times in PBS-T and then incubated with horseradish peroxidase-conjugated secondary antibodies (anti-rabbit IgG or anti-mouse IgG at 1:5000, from Agilent Technologies, CA, USA) diluted in blocking buffer for 4 hours at 4 °C. This step was followed by another 3 washes in PBS-T and finally chemiluminescent development using the ECL Western Blotting Substrate (Promega, WS, USA). The signal was captured with the BioRad ChemiDoc Imager (Bio-Rad Laboratories, CA, USA) and bands quantified using ImageJ (https://imagej.nih.gov/ij/).

### Immunoprecipitation

In preparation for immunoprecipitation, sepharose beads coated with the anti-myc-tag antibody (Cell Signaling Technology, MA, US) were blocked with 0.5% BSA in lysis buffer (150 mM NaCl, 50 mM Tris pH7.4, 1% Triton) for 1 hour at 4 °C. Then, cell lysate or growth medium containing myc-tagged BDNF was added to the beads and incubated at 4 °C for 2 hours, followed by 3 wash steps in lysis buffer and elution by boiling the beads in polyacrylamide gel loading buffer at 90 °C for 5 minutes.

### Furin cleavage assay

Myc-Tagged BDNF was expressed in HEK cells for 3 days followed by cell disruption in lysis buffer (1% Triton, 50 mM Tris, 150 mM NaCl) and centrifugation at 15,000 g for 10 min. The supernatant was kept on ice until further processing and anti-myc antibody coated sepharose beads (Cell Signaling Technology, MA, US) were blocked in 0.5% BSA followed by incubation with the cell lysate at 4 °C for 2 hours. Next, the beads with bound BDNF were washed 3 times with lysis buffer and then directly submitted to furin cleavage by incubation in 100 μl furin cleavage buffer (100 mM HEPES, 0.5% Triton, 1 mM CaCl2, 1 mM β-mercaptoethanol), with 1 unit furin (Sigma Aldrich, UK) at 25 °C for 5 hours. The cleaved protein was eluted by boiling the beads for 5 min at 90 °C in polyacrylamide gel loading buffer. The eluate was then analysed by Western blotting.

### Neurite Outgrowth Assay

PC12 cells co-transfected with either wild type or mutant TrkB constructs and a GFP expression construct were plated onto 96-well collagen-IV coated plates at the density of 1.0 ×10^5^ cells/ml. The next day the growth medium was replaced with serum free culture medium supplemented with 50 ng/ml of BDNF (either from R&D Systems or produced in house as outlined below). After 48 hours, the cells were treated with DAPI for 10 minutes and then photographed under a fluorescent microscope. Total length of neurites normalised to number of nuclei was measured using the ImageJ software (https://imagej.nih.gov/ij/). At least 300 cells were analysed per replicate.

### Growth Assay

PC12 cells transfected with either wild type or mutant TrkB were plated onto collagen-IV coated 96-well plates at a density of 1.0 ×10^6^ cells/ml. The next day the growth medium was replaced with serum free culture medium supplemented with 50 ng/ml of BDNF (RnD systems, UK), which was refreshed after 48 hours. After 96 hours total cell viability was quantify by XTT assay according to the manufacturer’s instructions (Roche, UK).

### KCl assay

PC12 cells transfected with c-terminally myc-tagged BDNF were grown in serum free RPMI medium for 24 hours, followed by treatment with 90 mM KCl for 15 minutes to trigger cellular depolarisation. The medium was collected and secreted BDNF captured by anti-Myc antibody coated sepharose beads, followed by immunoblotting to quantify protein levels.

### Production of recombinant mature BDNF

Mature recombinant BDNF was synthesised as previously described^36^; with modifications: Expression construct of Brain-derived neurotrophic factor - BDNF (residues 128–247, Uniprot: P23560) was amplified by PCR, using the forward primer 5′-TATATGGATCCCATAGTGACCCCGCCCGCCGTGGGGAGCTG-3′ and reverse primer 5′-ATATAAAGCTTAACGTCCACGTTTAATGGTCAGTGTAC-3′ and cloned into the pOP1 expression vector, using BamHI and HindIII restriction enzymes. The E183K mutant BDNF was generated by a two-step PCR protocol using overlapping oligonucleotides (forward primer5′-GAAGCAATATTTCTACAAGACCAAGTGTAATCCCATGGGT-3′ and reverse primer 5′-ACCCATGGGATTACACTTGGTCTTGTAGAAATATTGCTTC-3′). Upon confirmation of the plasmid sequence, it was transformed into *E. coli* strain BL21 (DE3) + pUBS520 for protein expression. Protein expression was performed at 37 °C using 2xYT media with 100 μg/ml of ampicillin and 25 μg/ml kanamycin for 3 hours after the addition of 400 μM isopropyl-1-thio-β-D-galactopyranoside (IPTG). Cells were harvested by centrifugation at 5,000 *g* for 20 min and stored at −20 °C.

### Protein refolding and purification

BDNF was refolded from inclusion bodies following a modified protocol used for pro-activin A^37^. Cell pellet from 1 L of bacterial culture was suspended in 50 mM Tris-HCl pH 8.0, 2 mM EDTA, 10 mM DTT (lysis buffer) with 0.5% (v/v) Triton-X 100 and lysed using the Emulsiflex C5 emulsiflier (Avestin, USA). The lysate was incubated with DNaseI for 20 min at room temperature to digest genomic DNA before centrifugation at 15,000 g for 20 min to pellet the insoluble inclusion bodies containing BDNF. The inclusion bodies were washed in lysis buffer with i) 0.5% Triton X-100, ii) with 1 M NaCl, and iii) with lysis buffer only. Purified inclusion bodies were re-suspended in 5 ml of 100 mM neutralized TCEP and protein denatured by addition of 15 ml of 8 M guanidine hydrochloride, 50 mM Tris-HCl pH 8.0, 5 mM EDTA. The sample was centrifuged at 15,000 g for 20 min and soluble BDNF was exchanged into 6 M urea and 20 mM hydrochloric acid. Refolding was started by adding the denatured BDNF into cold solution of 100 mM Tris-HCl pH 8.0, 1 M pyridinium propyl sulfobetaine, 250 mM NaCl, 0.2 mM cystine and 2 mM cysteine with vigorous stirring. The refolding solution was kept at 7 days at 4 °C, after which it was clarified by filtering and loaded onto Source 30 S cation exchange column (GE Healthcare, USA), equilibrated with 50 mM Tris pH 8.0, 50 mM NaCl. Protein was eluted with a linear gradient to 1 M NaCl in 30 column volumes and peak fractions containing BDNF pooled. The sample was acidified and purified further by reverse-phase chromatography (RPC) using HiChrom-5C8-25002 column (Hichrom, UK) equilibrated in 10% acetonitrile and 0.1% trifluoroacetic acid (TFA) and sample eluted with a gradient of increasing acetonitrile. Purity was confirmed by SDS-PAGE analysis of reduced and non-reduced samples and correct mass (assuming fully disulfide-linked protein) confirmed by LC-MS analysis. Pure protein was dried under vacuum and stored at −80 °C.

### Aggregate formation and BDNF treatment

Hypothalamic neurons from pluripotent stem cells (hPSC) were generated according to previously published methods^38-9^. The cell line used was HUES9 ES (Passage 32-40, Harvard University, RRID: CVCL_0057)^38,39^. The cells were detached on day 32 followed by aggregation in N2B27 media (described in^38^) in U-bottom shaped 96-well plates (Lipidure-coat plate A-U96; Amsbio, UK) at a density of 15,000 cells per well. Aggregates were allowed to form for 3 days, and then transferred to the centre of each well of a 24-well plate (Integrated BioDiagnostics, UK) coated with Geltrex (Thermo Fisher Scientific, USA). After 24 hours, aggregates had adhered to the 24-well plates, and were then treated with N2B27 containing WT or Mutant (E183K) BDNF at the following concentrations: 0 pg/mL, 5 pg/mL, 20 pg/mL, 100 pg/mL, 500 pg/mL, 2 ng/mL, 10 ng/mL, 50 ng/mL (n = 3-6 aggregates per BDNF concentration). Aggregates were fed daily with this media (3/4 media change) for 8 days. Aggregate formation was quantified by Immunocytochemistry (see below).

### Monolayer formation and BDNF treatment

Hypothalamic neurons from induced pluripotent stem cells (iPSC) were generated according to previously published methods^38^ and were detached on day 32 followed by plating as monolayers at a density of 25,000 cells per cm^2^ on a 24-well plate (Integrated BioDiagnostics, UK) coated with Geltrex (Thermo Fisher Scientific, USA). 3-days after plating, the monolayers were treated with N2B27 containing WT or Mutant (E183K) BDNF at the following concentrations: 0 pg/mL, 5 pg/mL, 20 pg/mL, 100 pg/mL, 500 pg/mL, 2 ng/mL, 10 ng/mL, 50 ng/mL (n = 3 wells). Monolayers were fed daily with this media (3/4 media change) for 8 days.

### Immunocytochemistry

Aggregates and monolayers were fixed at room temperature for 10 mins in 4% paraformaldehyde in PBS. After brief washes in TBS, cells were incubated overnight at 4 °C in primary antibody diluted in TBS 0.1% Triton X-100 with 5% Normal Donkey Serum. Primary antibodies used for the study were: anti-POMC (A1H5, mouse, 1:5000; a kind gift from Anne White, University of Manchester), anti-MAP2 (1:2000; Abcam, UK), and anti-Tuj1 (1:2000; BioLegend, UK) anti-PSD-95 (clone K28/43, 1:1000; Upstate biochemical), anti-GluR1 (extracellular, 1:1000; AGC-004, AbCAM), anti-vGlut1, (1:1000, Clone N28/9; NeuroMAB). The following day the primary antibody was washed out with TBS and cells were treated at room temperature for 2 hours with species-specific Alexa Fluor conjugated secondary antibodies (Thermo Scientific, USA) diluted to 1:500 in TBS 0.1% Triton X-100 with 5% Normal Donkey Serum. After washing out the secondary antibody, the cells were treated with DAPI for 5 min at room temperature and stored in TBS with 0.1% Sodium Azide.

### Imaging acquisition

Monolayer and aggregates were imaged on a Zeiss LSM 700 confocal microscope (Zeiss AG, Germany), or on an INCell Analyser 2200 automated microscope (GE Healthcare, USA). Image analysis was performed on ImageJ (https://imagej.nih.gov/ij/). For neurite length measurements, neurites from individual, spatially isolated POMC neurons were traced in the POMC and Tuj1 channels using the Freehand line and then measure using the measurement tools. Total neurite lengths were then determined by summing the lengths of neurites for each neuron. For BDNF-induced POMC neuron migration POMC-positive cells outside the aggregate body - defined for each aggregate individually by DAPI, MAP2 and Tuj1 expression - were counted and normalised to area.

### Use of rats as a model organism

All methods were carried out in accordance with relevant guidelines and regulations for use of animals in the study. All of the animal studies presented in this manuscript were performed at Washington State University (WSU), and were conducted under humane conditions, with appropriate regard for animal welfare. WSU is a registered research facility with the US Department of Agriculture (USDA) and is committed to comply with the Guide for the Care and Use of Laboratory Animals (Department of Health and Human Services), the provisions of the Animal Welfare Act (USDA) and all applicable federal and state laws and regulations. In addition, WSU is an AAALAC accredited institution. WSU has established an “Animal Care and Use Committee” to ensure compliance with all applicable federal and state regulation for the purchase, transportation, housing and research use of animals. WSU has filed an appropriate Assurance and Compliance with the office of Laboratory Animal Welfare (OLAW) at NIH. All experimental protocols relating to the use of neurons from animals for hippocampal cultures were approved by the Washington State University Institutional Animal Care and Use Committee under approved protocols 03717-019 and 04409-006.

### Hippocampal Cell Culture Preparation and transfection

Hippocampal neuronal cultures were prepared as previously described^40^. Primary hippocampal cultures were transfected with Lipofectamine 2000 (Life Technologies, USA). Native media was collect before transfection and replaced with warm growth media. Lipofectamine 2000 and experimental DNA plasmids (0.5 µg/well for 24-well plates) were added to cells and incubated for 30 min. The media was then aspirated and replaced with native media.

### Hippocampal Spine Quantification

Hippocampal cultures were transfected with designated DNA constructs and Clover-βactin to allow for visualisation of dendritic spine density and morphology^41^. Confocal fluorescent images were obtained using the Metamorph software (Molecular Devices, USA) and a Leica DMI6000 SD confocal microscope equipped with a Yokogawa CSU-X1 spinning disk. Dendritic spine and filopodia density was measured on primary and secondary dendrites at a distance of at least 100 μm from the soma. Two to five dendrites, each at least 50 μm in length, from at least 25 neurons were analysed for each data point reported. Each experiment was repeated at least three times using independent culture preparations. Dendrite length was determined using ImageJ 1.41 (National Institutes of Health, Bethesda, MD) and the neurite tracing program Neuron J^41^. Spines and filopodia were manually counted.

### BDNF vesicle co-localization and density measurements

Hippocampal neurons were transfected as described above with soluble Blue Flourescent Protein (BFP) to visualise dendrites and axons and either RFP or GFP tagged wtBDNF or E183K-BDNF. Confocal fluorescent images were obtained as in 2.7. High resolution images of dendritic processes where obtained from primary and secondary dendrites at a distance of at least 100 μm from the soma. Two to five dendrites, each at least 50 μm in length were analysed. High resolution images of axon processes where obtained 1000 μm from the soma. Two to five axon segments, each at least 50 μm in length were analysed. 600–1000 vesicles were analysed for wt-BDNF and mutant-BDNF vesicle co-localisation. Axonal and dendritic vesicle density was calculated from at least 25 neurons from three independent cultures.

### Whole-Cell Recordings

Patch-clamp experiments were performed - as reported previously^40^ - on Clover-βactin-transfected cultured hippocampal neurons with PBS (vehicle control) or BDNF pretreatment. Recordings were made on DIV10 to DIV11. The culture medium was exchanged by an extracellular solution containing 140 mM NaCl, 2.5 mM KCl, 1 mM MgCl_2_, 3 mM CaCl_2_, 25 mM glucose, and 5 mM HEPES; pH was adjusted to 7.3 with KOH, and osmolality was adjusted to 307–310 mOsM. Cultures were allowed to equilibrate in a recording chamber mounted on an inverted microscope (IX-71; Olympus Optical) for 30 min before recording. Transfected cells were visualised with fluorescence (Olympus Optical). Recording pipettes were pulled (P-97 Flaming/Brown micropipette puller; Sutter Instrument Company, Novato, CA) from standard-wall borosilicate glass without filament (o.d. = 1.5 mm; Sutter Instrument Company). The pipette-to-bath d.c. resistance of patch electrodes ranged from 4.0 to 5.2 MΩ, and they were filled with an internal solution of the following composition: 25 mM CsCl, 100 mM CsCH3O3S, 10 mM phospho-creatine, 0.4 mM EGTA, 10 mM HEPES, 2 mM MgCl2, 0.4 mM Mg-ATP, and 0.04 mM Na-GTP; pH was adjusted to 7.2 with CsOH, and osmolality was adjusted to 296 to 300 mOsM. Miniature EPSCs (mEPSCs) were isolated pharmacologically^40^ by blocking GABA_A_ receptors with picrotoxin (100 μM; Sigma-Aldrich), blocking glycine receptors with strychnine (1 μM; Sigma-Aldrich), and blocking action potential generation with tetrodotoxin (500 nM; Tocris Bioscience, Ellisville, MO). Recordings were obtained using a Multiclamp 700B amplifier (Molecular Devices, Sunnyvale, CA). Analog signals were low-pass Bessel-filtered at 2 kHz, digitized at 10 kHz through a Digidata 1440 A interface (Molecular Devices), and stored in a computer using Clampex 10.2 software (Molecular Devices). The membrane potential was held at -70 mV at room temperature (25 °C) during a period of 0.5 to 2 h after removal of the culture from the incubator. Liquid junction potentials were not corrected. Data analysis was performed using Clampfit 10.2 software (Molecular Devices) and Mini-Analysis 6.0 software (Synaptosoft, Decatur, GA). The criteria for a successful recording included an electrical resistance of the seal between the outside surface of the recording pipette and the attached cell >2 GΩ and neuron input resistance >240 MΩ. The mEPSCs had a 5-min recording time.

## Supplementary information

Supplementary Information.

Supplementary Figure Legends

Supplementary Figure S1

Supplementary Figure S2

Supplementary Figure S3

## Data Availability

No datasets were generated or analysed during the current study.
