# Supplementary material for: Human BDNF/TrkB variants impair hippocampal synaptogenesis and associate with neurobehavioural abnormalities
Source: Sci Rep. 2020 Jun 3;10:9028. doi: 10.1038/s41598-020-65531-x (PMC7270116; doi:10.1038/s41598-020-65531-x)
Supplement: Supplementary file 1 — Supplementary Information. [file 41598_2020_65531_MOESM1_ESM.docx]

**Human BDNF/TrkB variants impair hippocampal synaptogenesis and associate with neurobehavioral abnormalities**

Takuhiro Sonoyama^1,7^, Lukas K.J. Stadler^1,7^, Mingyan Zhu^2^, Julia M. Keogh^1^, Elana Henning^1^, Fuki Hisama^3^, Peter Kirwan^1^, Magdalena Jura^1^, Beata K. Blaszczyk^4^, David C. DeWitt^2^, Bas Brouwers^1^, Marko Hyvönen^4^, Inês Barroso^5,6^, Florian T. Merkle^1^, Suzanne M. Appleyard^2^, Gary A. Wayman^2,8,^*, I. Sadaf Farooqi^1,8,*^.

^1^University of Cambridge Metabolic Research Laboratories and NIHR Cambridge Biomedical Research Centre, Wellcome Trust-MRC Institute of Metabolic Science, Addenbrooke's Hospital, Cambridge, UK; ^2^Integrative Physiology and Neuroscience, College of Veterinary Medicine, Washington State University, Pullman, Washington, USA; ^3^Department of Medicine (Medical Genetics), University of Washington School of Medicine, Seattle, Washington, USA; ^4^Department of Biochemistry, 80 Tennis Court Road, CB2 1QW, University of Cambridge, UK; ^5^MRC Epidemiology Unit, Addenbrooke's Hospital, Cambridge, UK; ^6^Wellcome Sanger Institute, Cambridge, UK.

^7^these authors contributed equally; ^8^these authors contributed equally; *corresponding authors: [isf20@cam.ac.uk](mailto:isf20@cam.ac.uk) and [waymang@wsu.edu](mailto:waymang@wsu.edu)

**Supplementary Information – Full Western Blot Images**

**Supplementary Information – Full Western Blot Images**

**Figure 1**

**C.**


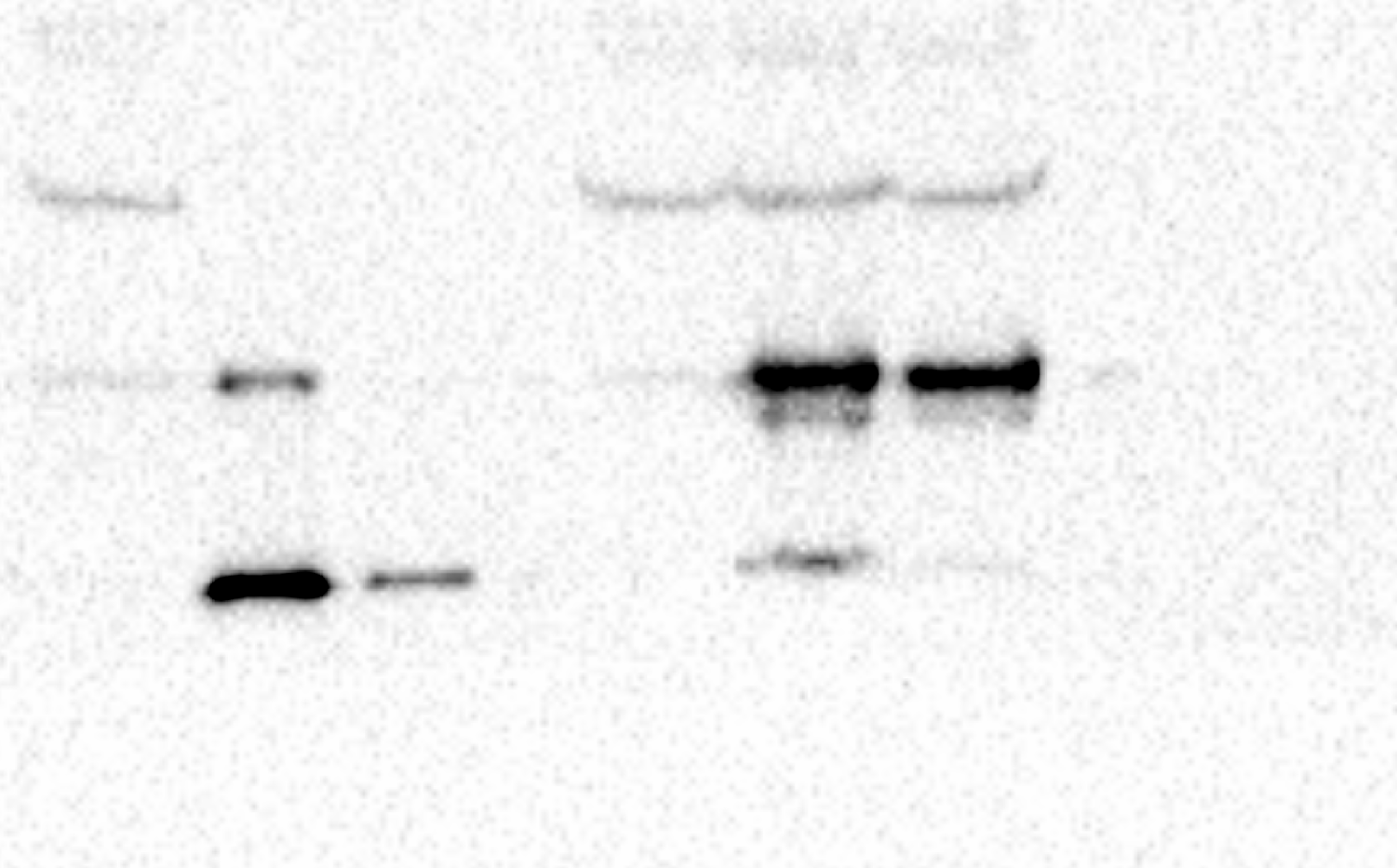


ii. i.


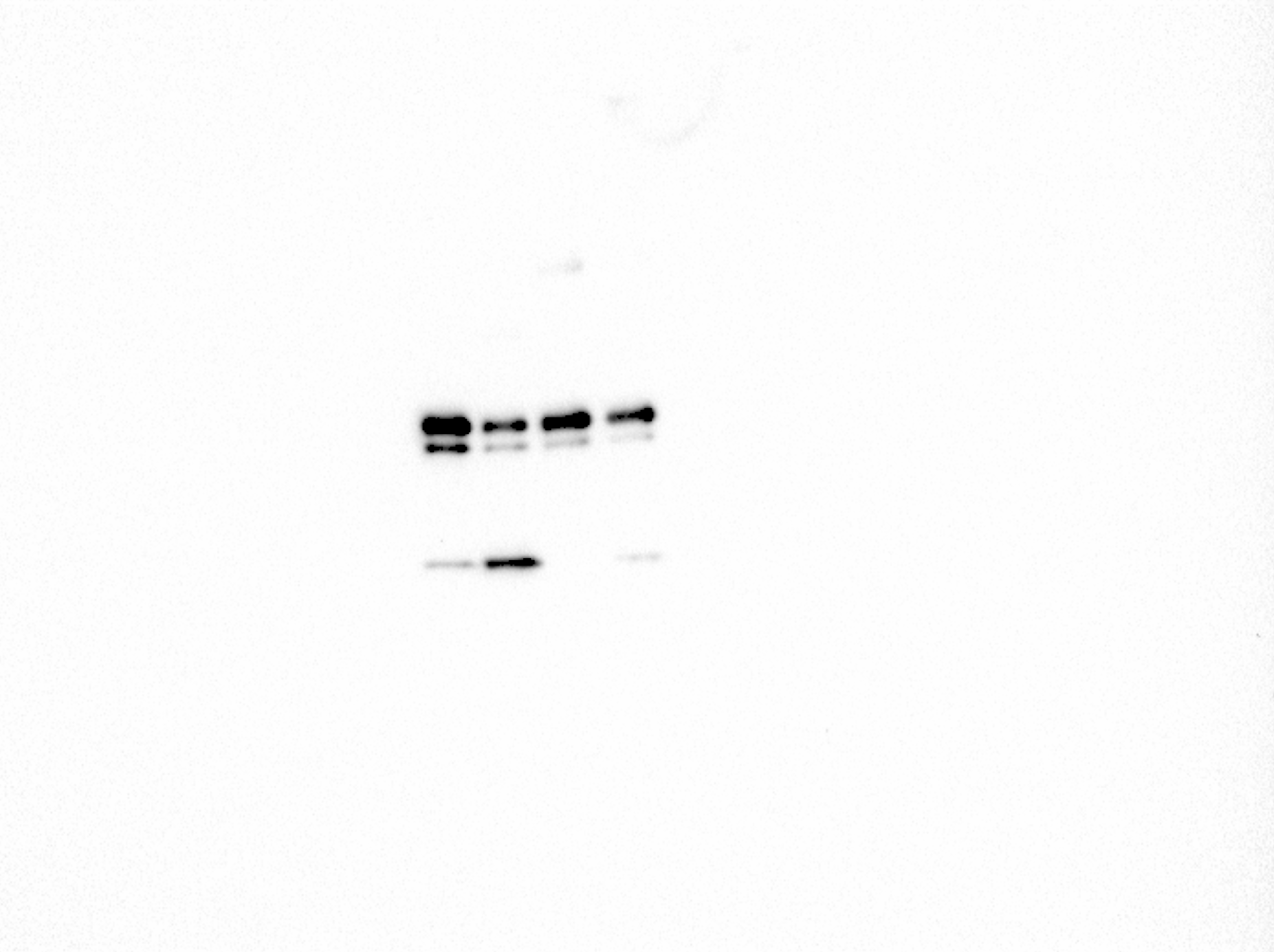


E.

F.


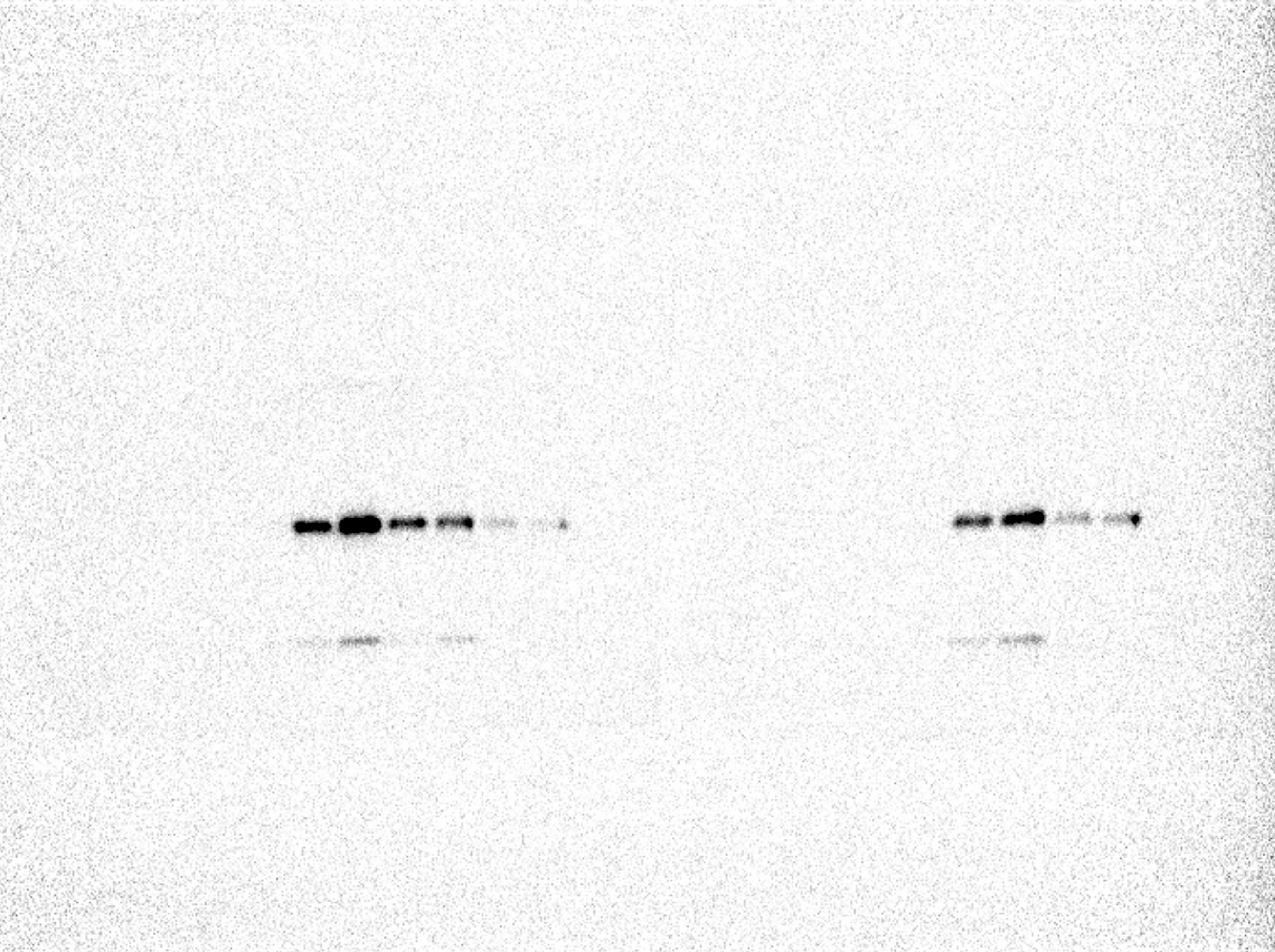


Figure 2


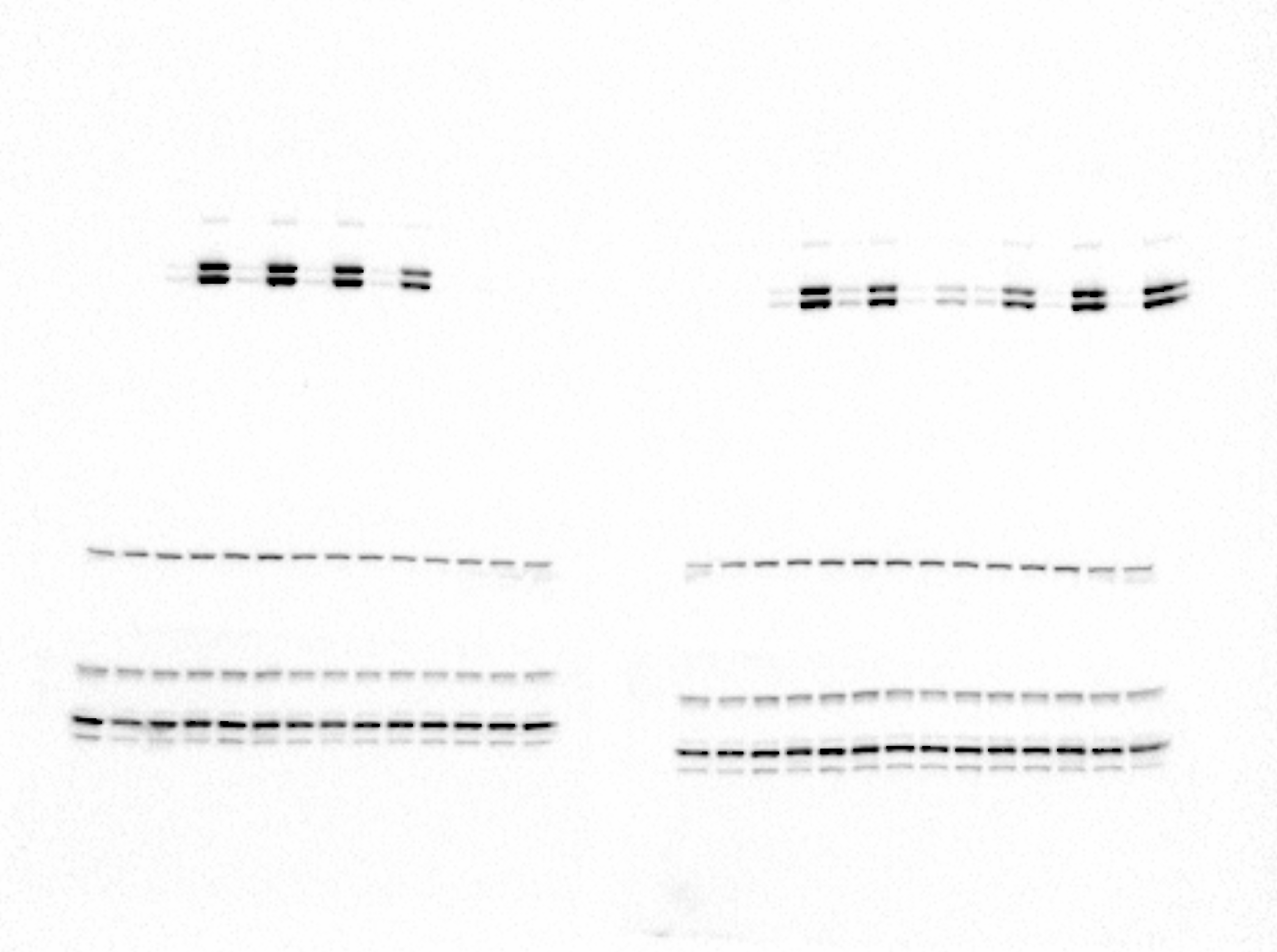


pERK

PLCy

AKT

ERK


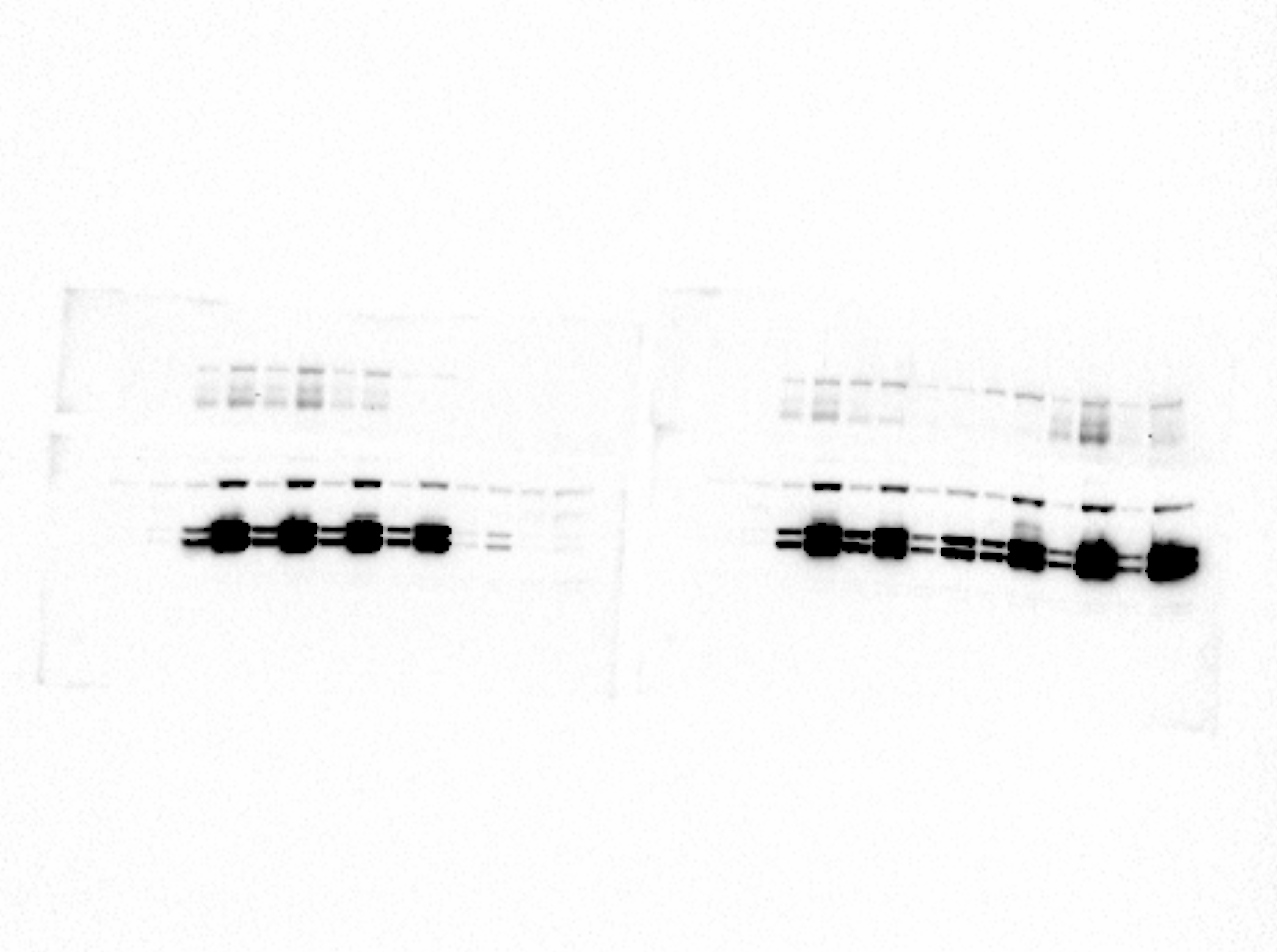


pPLCy

pAKT
