# Supplementary material for: Human BDNF/TrkB variants impair hippocampal synaptogenesis and associate with neurobehavioural abnormalities
Source: Sci Rep. 2020 Jun 3;10:9028. doi: 10.1038/s41598-020-65531-x (PMC7270116; doi:10.1038/s41598-020-65531-x)
Supplement: Supplementary file 2 — Supplementary Figure Legends [file 41598_2020_65531_MOESM2_ESM.docx]

**Human BDNF/TrkB variants impair hippocampal synaptogenesis and associate with neurobehavioral abnormalities**

Takuhiro Sonoyama^1,7^, Lukas K.J. Stadler^1,7^, Mingyan Zhu^2^, Julia M. Keogh^1^, Elana Henning^1^, Fuki Hisama^3^, Peter Kirwan^1^, Magdalena Jura^1^, Beata K. Blaszczyk^4^, David C. DeWitt^2^, Bas Brouwers^1^, Marko Hyvönen^4^, Inês Barroso^5,6^, Florian T. Merkle^1^, Suzanne M. Appleyard^2^, Gary A. Wayman^2,8,^*, I. Sadaf Farooqi^1,8,*^.

^1^University of Cambridge Metabolic Research Laboratories and NIHR Cambridge Biomedical Research Centre, Wellcome Trust-MRC Institute of Metabolic Science, Addenbrooke's Hospital, Cambridge, UK; ^2^Integrative Physiology and Neuroscience, College of Veterinary Medicine, Washington State University, Pullman, Washington, USA; ^3^Department of Medicine (Medical Genetics), University of Washington School of Medicine, Seattle, Washington, USA; ^4^Department of Biochemistry, 80 Tennis Court Road, CB2 1QW, University of Cambridge, UK; ^5^MRC Epidemiology Unit, Addenbrooke's Hospital, Cambridge, UK; ^6^Wellcome Sanger Institute, Cambridge, UK.

^7^these authors contributed equally; ^8^these authors contributed equally; *corresponding authors: [isf20@cam.ac.uk](mailto:isf20@cam.ac.uk) and [waymang@wsu.edu](mailto:waymang@wsu.edu)

# **Supplementary Figure legends**

**Figure S1. Functional characterization of mutant (E183K) BDNF.** A. The density of vesicles carrying WT or WT and mutant BDNF were quantified by confocal microscopy in dendrites and axons of cultured primary rat hippocampal neurons using the same cells as shown in Figure 1D (data point = one dendrite/axon analysed). *p<0.05, student’s t-test. B. Recombinantly expressed, mature BDNF was analysed for purity and size by polyacrylamide gel electrophoresis, with (+βME) or without (-βME) reducing agent. C. TrkB-expressing PC12 cells were stimulated with synthetic WT/mutant BDNF for 10 minutes and activation (phosphorylation) of downstream signalling cascades measured by Western blotting. D. Left panel shows migration of human iPSC-derived POMC neurons from central cell aggregates following treatment with WT/mutant mature BDNF (scale bar: 100 μm); quantified data shown in the right panel (data point = mean of independent replicates). *p<0.05, student’s t-test.

**Figure S2. Functional characterization of TrkB mutants.** A. Expression levels of all TrkB mutants measured by Western blotting, by probing for the C-terminally fused myc epitope tag. B. PC12 cells were transiently transfected with WT/mutant TrkB as well as GFP and stimulated with recombinant BDNF for 48 h. Neurite length was measured by fluorescent microscopy and quantified as length per nucleus (See Figure 2C). Scale bar: 50 μm. C. PC12 cells were transiently transfected with WT/ mutant TrkB and stimulated in serum free medium with recombinant BDNF for 96 hours. Total cell viability was measured via XTT assay and expressed relative to WT (data point = mean of independent replicates). *p<0.05, student’s t-test.

**Figure S3. Functional characterization of TrkB mutants in neurons.** The average interval (msec) for recorded mEPSCs before (-) and after (+) BDNF stimulation is shown. **/* indicates significantly (p<0.01/0.05) reduced interval upon BDNF stimulation; One way ANOVA-Tukey, F=15.93.
