# Supplementary figures and images for: Human BDNF/TrkB variants impair hippocampal synaptogenesis and associate with neurobehavioural abnormalities
Source: Sci Rep. 2020 Jun 3;10:9028. doi: 10.1038/s41598-020-65531-x (PMC7270116; doi:10.1038/s41598-020-65531-x)

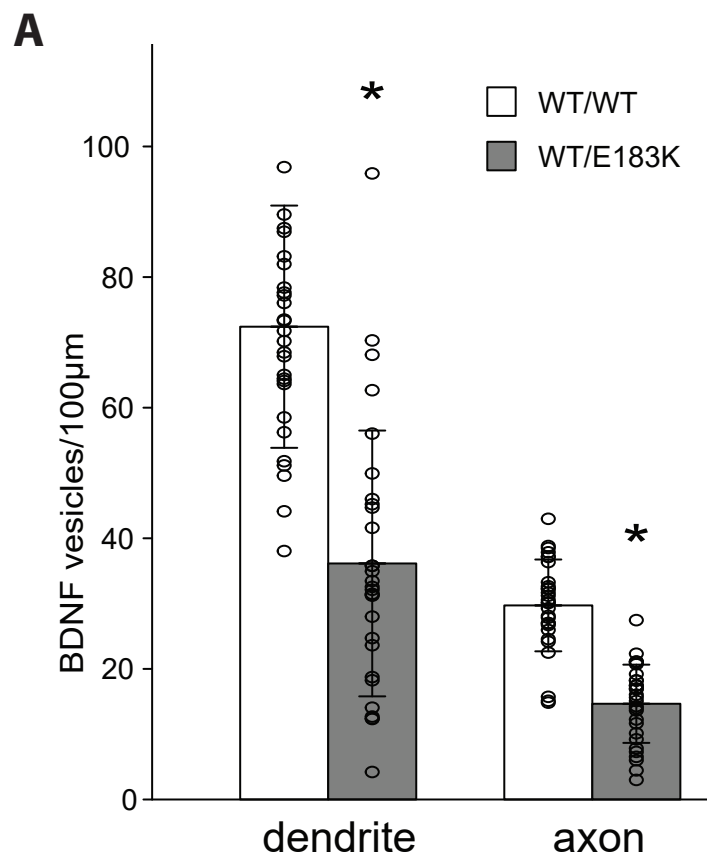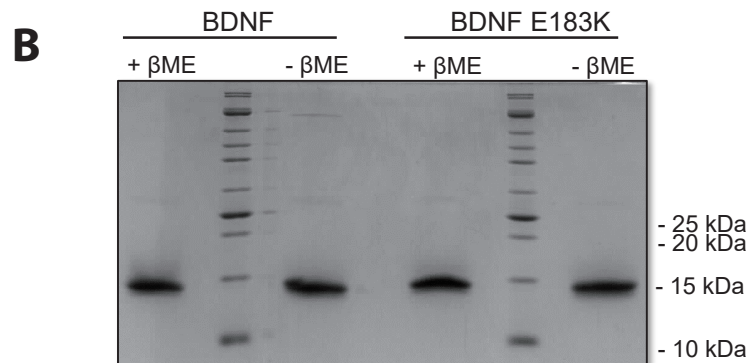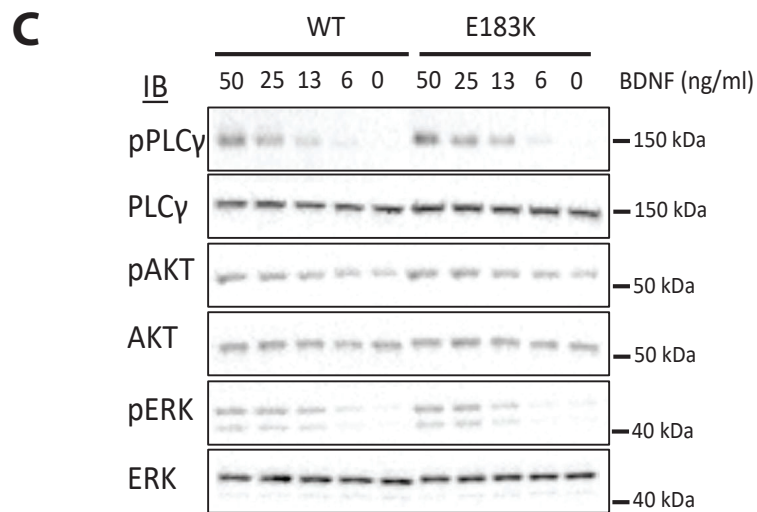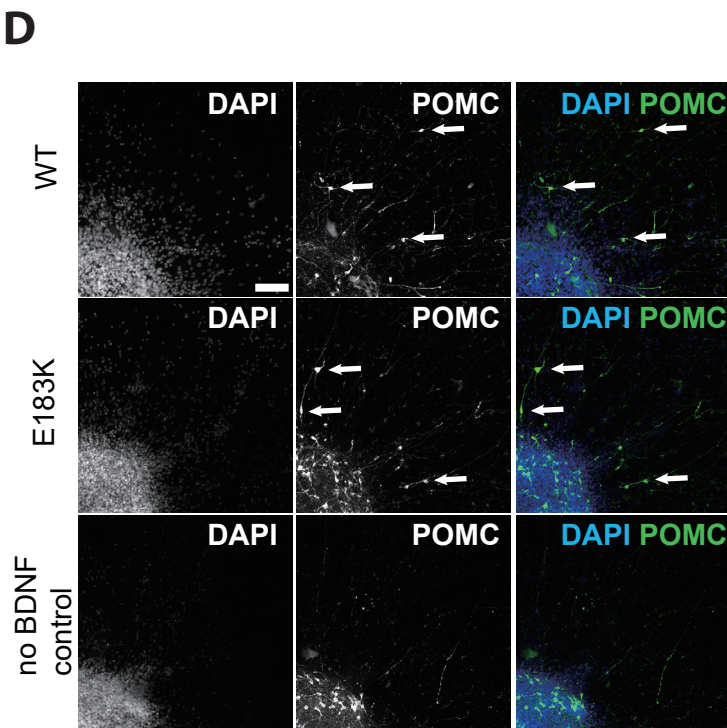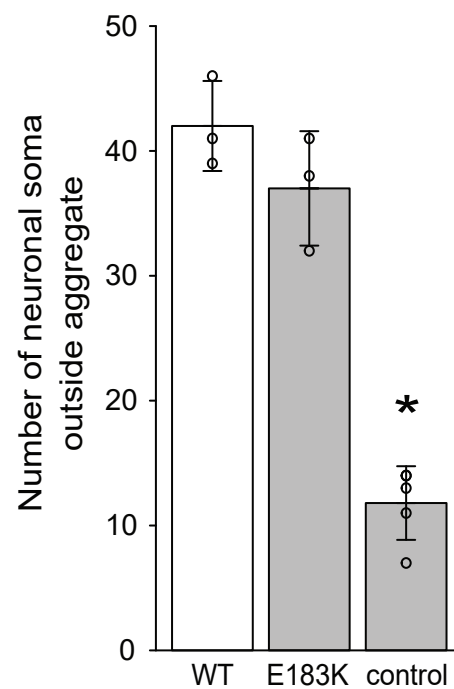

Supplement: Supplementary file 3 — Supplementary Figure S1 [file 41598_2020_65531_MOESM3_ESM.pdf]

**A**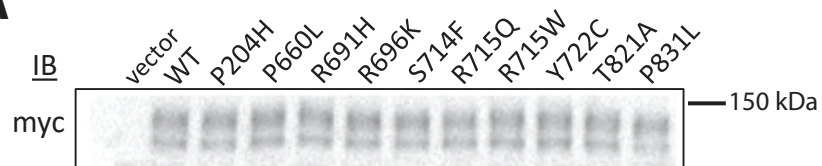**B**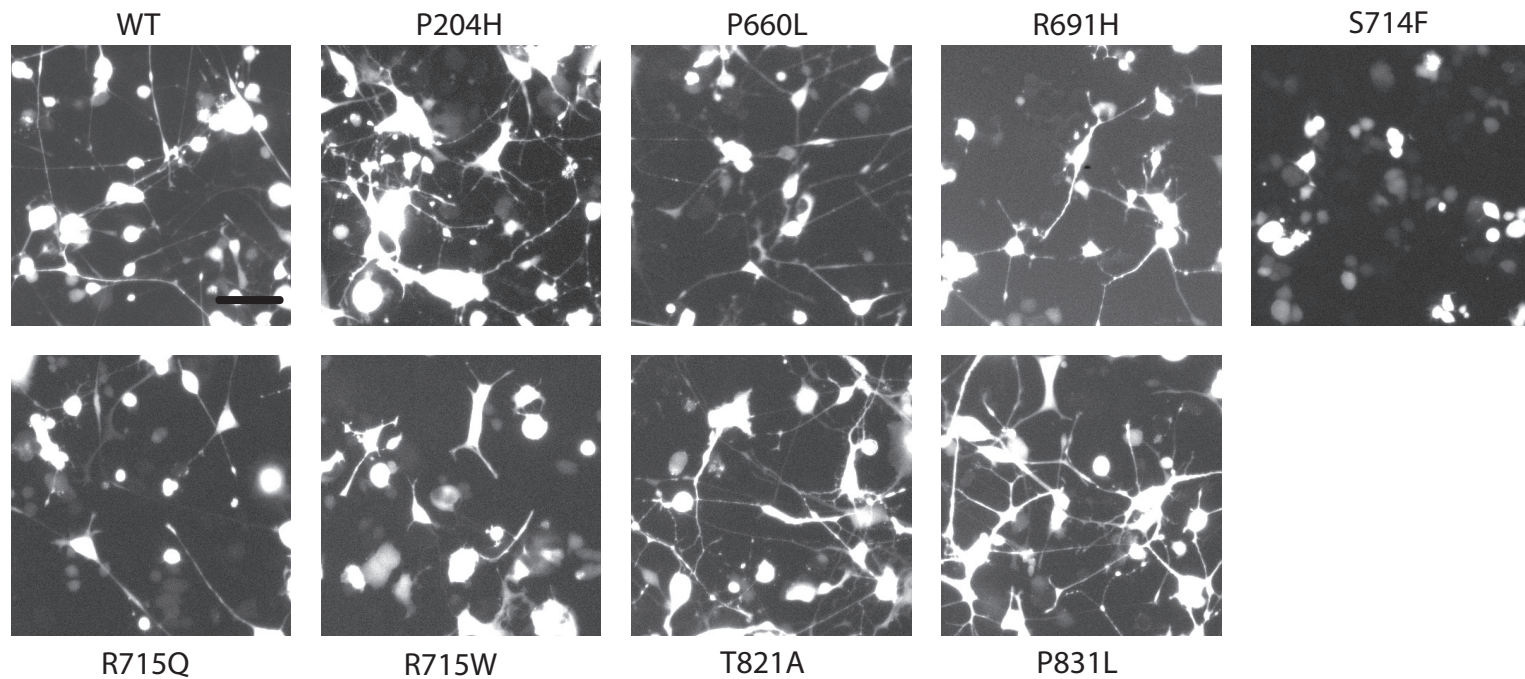**C**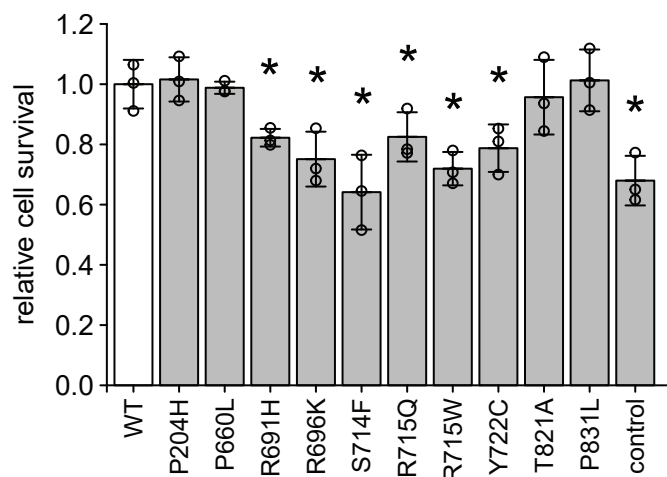

Supplement: Supplementary file 4 — Supplementary Figure S2 [file 41598_2020_65531_MOESM4_ESM.pdf]

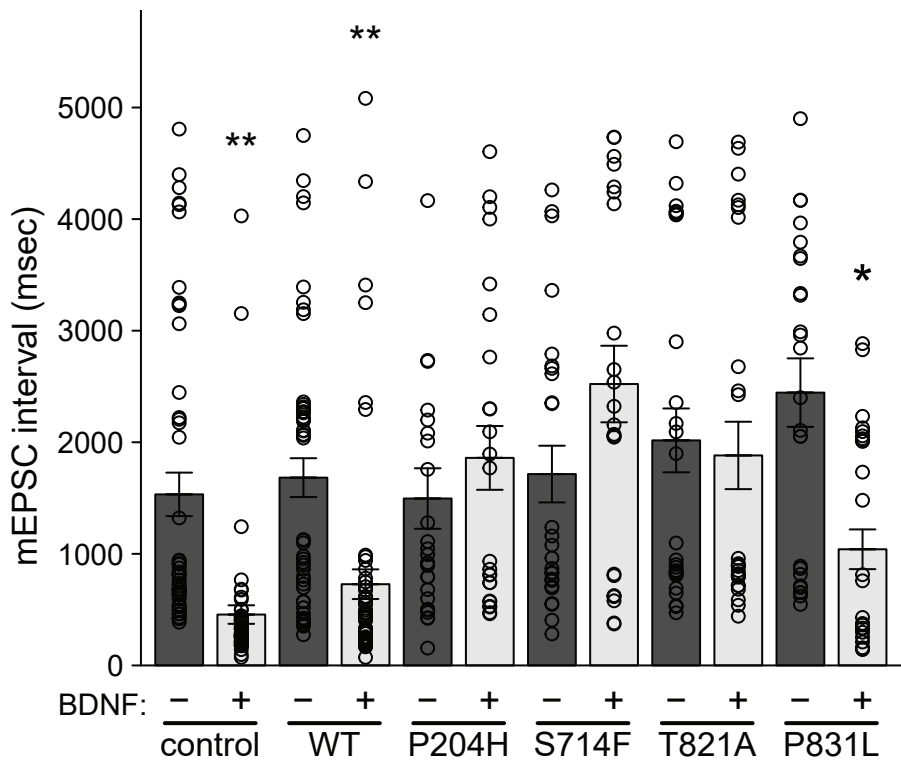

Supplement: Supplementary file 5 — Supplementary Figure S3 [file 41598_2020_65531_MOESM5_ESM.pdf]
